# Antinociceptive Effect of Hydroalcoholic Extract of Iranian Green tea in the Formalin Test in Rats

**Published:** 2013-02-13

**Authors:** Ardeshir Arzi, Behnam Ghorbanzadeh, Zahra Nazari Khorasgani

**Affiliations:** 1 Department of Pharmacology and Toxicology, School of Pharmacy, Physiology Research Center, Jundishapur University of Medical Sciences, Ahvaz, IR Iran; 2 Department of Pharmacology and Toxicology, School of Pharmacy, Jundishapur University of Medical Sciences, Ahvaz, IR Iran

**Keywords:** Green Tea, Antinociception, Formalin Test, Rat

## Abstract

**Background:**

Tea *(Camellia sinensis)* has been utilised, since time immemorial, as a beverage possessing encouraging health benefits. Little scientific evidence exists in literature on the effect of this plant on pain.

**Objectives:**

To investigate the antinociceptive activity of Iranian green tea extract.

**Materials and Methods:**

The hydroalcoholic extract was administered to male Wistar rats. Formalin paw test was used to evaluate the antinociceptive activity. Plant extract (25, 50, 100 and 200 mg/kg, i.p.) (n = 6 for each group) or vehicle (n = 6) was administered 30 min before the subplantar formalin injection.

**Results:**

The extract caused a significant dose-related (50, 100, 200 mg /kg, i.p.) inhibition of the first phase and onset of chronic phase (200 mg /kg, i.p.) of formalin induced nociception. The results showed that the pre-treatment of rats with naloxone (1 mg/kg, i.p.) significantly (P < 0.001) reversed antinociception by Green tea extract (GTE) (200 mg/kg, i.p.) in the inflammatory phase and had no effect on phase 1.

**Conclusions:**

These results indicate that GTE produces dose-related antinociception in chemical pain model and one of its possible mechanisms involves opioid pathways.

## 1. Background

Pain is the most common reason for patients to seek advice from health professionals. It is one of the most frequent presenting symptoms of different pathologies and represents important medical and economic costs for the community (1). Current analgesic therapies, despite their proven efficacy in alleviating symptoms and providing pain relief, all have considerable side effects including gastrointestinal problems, renal damage, respiratory depression, emesis, and tolerance and/or addiction (2). In addition, many pain sufferers are not satisfied with their pain care and this makes the search for new analgesics that can treat pain more effectively, an important challenge to drug research. Medicinal plants are believed to be important sources of new chemical substances with potential therapeutic efficacy. Considering that the most important analgesic prototypes (e.g., salicylic acid and morphine) were originally derived from plant sources. The study of plant species traditionally used as analgesics should still be seen as a useful research strategy in the search of new analgesics.

Tea *(Camellia sinensis)* has been utilised, since time immemorial, as a beverage possessing encouraging health benefits. Green tea is rich in flavonoids. The clinical and experimental studies on human being and animals have shown that green tea has antiinflammatory (3, 4), antibacterial (5), anticarcinogenic effects (6), and it also lowers plasma lipids and glucose levels (7, 8). These effects have largely been attributed to the most prevalent polyphenol contained in green tea, the catechin or flavanol (-) epigallocatechin-3-gallate (EGCG).There is a variation between bioactive components of plants that may be mainly attributed to the different sample extraction methods and different geographic origins of herbal tea. In addition to intrinsic factors, the flavonol content in plants is strongly influenced by extrinsic factors such as variations in plant type and growth, season, climate, degree of ripeness, food preparation and processing (9).

## 2. Objectives

In Iran, tea is cultivated in northern area and is the most popular beverage after water. Despite the large number of pharmacological studies on tea, carried out worldwide, there is little scientific evidence in literature on the effect of this plant on experimental pain. The present study, therefore, aimed to evaluate the possible ant nociceptive and related mechanism of hydroalcoholic extract of Iranian green tea in animal models. The current study will help to substantiate the traditional uses of Iranian green tea as well as providing an alternative to current analgesics.

## 3. Materials and Methods

### 3.1. Plant Material and Preparation of the Extract

Iranian green tea was purchased from the market (Refah Co.). To prepare hydroalcoholic extract, powdered plant (100 g) was macerated by 1500 ml of ethanol 70% (v/v) for 72 h. The extract was then shaken, filtered and the solvent was removed in a vacuum evaporator to obtain semi-solid extract and then it was placed in an oven in 60°C for 72 h (10).

### 3.2. Animals

Male Wistar rats weighing 150–200 g were obtained from a random bred colony in the animal house of Ahvaz Jundishapur University of Medical Sciences. Animals were housed in standard cage, on 12 h light/dark cycle; and air temperature was maintained at 22 ± 2 ◦C. Experiments reported in this study were carried out in accordance with local guidelines for the care of laboratory animals of Ahvaz Jundishapur University of Medical Sciences.

### 3.3. Formalin Test

The formalin test was carried out in a 30 × 30 × 30 cm clear plastic chamber with a mirror placed under the floor to allow an unobstructed view of the paws. Behavior was rated for 0–5 min (first phase), 15–60 min (second phase) after subplantar injection of 50 μl formalin (2.5%) into the right hind paw. To analyze data, the second phase was also subdivided into phase 2A (15–35min) and phase 2B (35–60 min), representing the onset and offset of the late phase, respectively, as described previously (11). Pain score was given originally described by Dubuisson and Dennis (1977): 0 = normal weight bearing on the injected paw; 1 = limping during locomotion or resting the paw lightly on the floor; 2 = elevation of the injected paw so that at most the nail touch the floor; and 3 = licking, biting or grooming the injected paw (12).

Hydroalcoholic extract (5ml/kg) was given i.p. (30 min) prior to formalin injection. Control animals received vehicle (5 mL/kg of normal saline). Morphine (2.5 mg/kg, i.p.) and aspirin (300mg/kg, i.p.) pretreated animals were included in the study for comparison.

### 3.4. Participation of Opioid System

To investigate the possible participation of the opioid system on the antinociceptive effect of GTE, the rats were pre-treated with naloxone (1.0 mg/kg, i.p.), and after 15 min, the animals received an injection of GTE (200 mg/kg, i.p.). The nociceptive response was recorded immediately after formalin injection.

### 3.5. Statistical Analysis

All the data were expressed as mean ± SEM. Since collected data were normally distributed according to the Kolmogorov and Smirnov Normality Test, parametric statistics was used. The effect of the green tea extract on the pain tests was evaluated by performing one-way ANOVA, followed by Tukey test. The pain scores observed were converted to percentage of maximum possible effect (%MPE) as follows:

%MPE = [ (E_Max_ – E) / (E_Max_ - E_Min_) ] ×100

Where *E*_*Max*_ and *E* are the mean of pain scores before (control) and after the treatment, respectively, and *E*_*Min*_ is minimum of pain score.

ED_50_ (dose responsible for 50% of the maximal effect) was determined by using an iterative computer least-squares method, with the following nonlinear regression equation: 

Y= (a + (b - a)) / (1 + 10 ^(^^logED^_50_^-X)^)

Where X is the logarithm of the dose and Y is the response. Y starts at a (the bottom) and goes to b (the top) with a sigmoid shape. GraphPad Prism for Windows, version 5.04 (GraphPad Software, San Diego, CA, USA) was used for all statistical analyses and ED50 determination. A P < 0.05 was considered statistically significant for analyses.

## 4. Results

Injection of formalin (2.5%, 50 μL) into the ventral surface of the right hind paw evoked a characteristic biphasic nociceptive response in animal models as previously reported (13). It consisted of an initial intense response to pain beginning immediately after formalin injection and rapidly decaying within 10 min after formalin injection (first/neurogenic phase) and then followed by a slowly rising but longer lasting response (second/inflammatory phase) from 10 to 60 mins after formalin injection (14).

**Figure 1:**
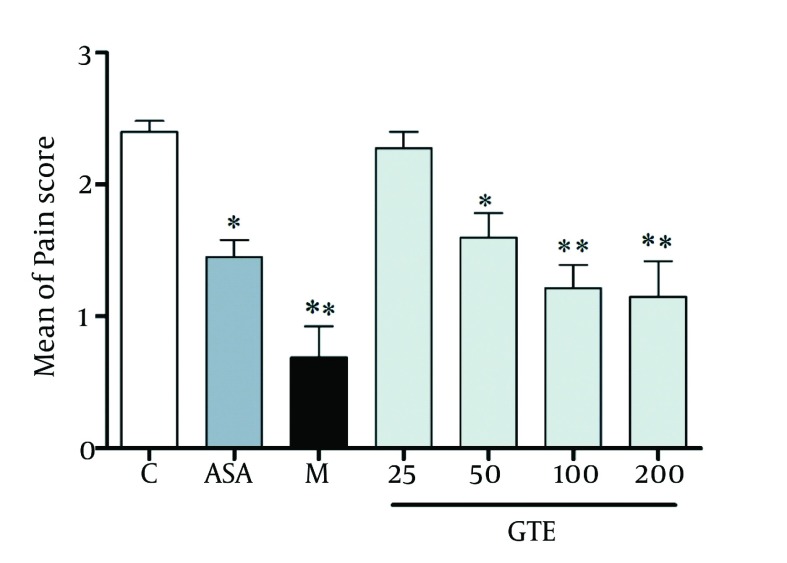
Effect of GTE (25-200 mg/kg, i.p.), Morphine (2.5mg/kg, i.p.) and Aspirin (300mg/kg, i.p.) on First Phases of the Formalin-induced Nociception Test in rat. Each Column Represents Mean ± SEM (n = 6). *P < 0.05 and ** P <0.01 Compared to Control (Treated With Vehicle).

**Figure 2:**
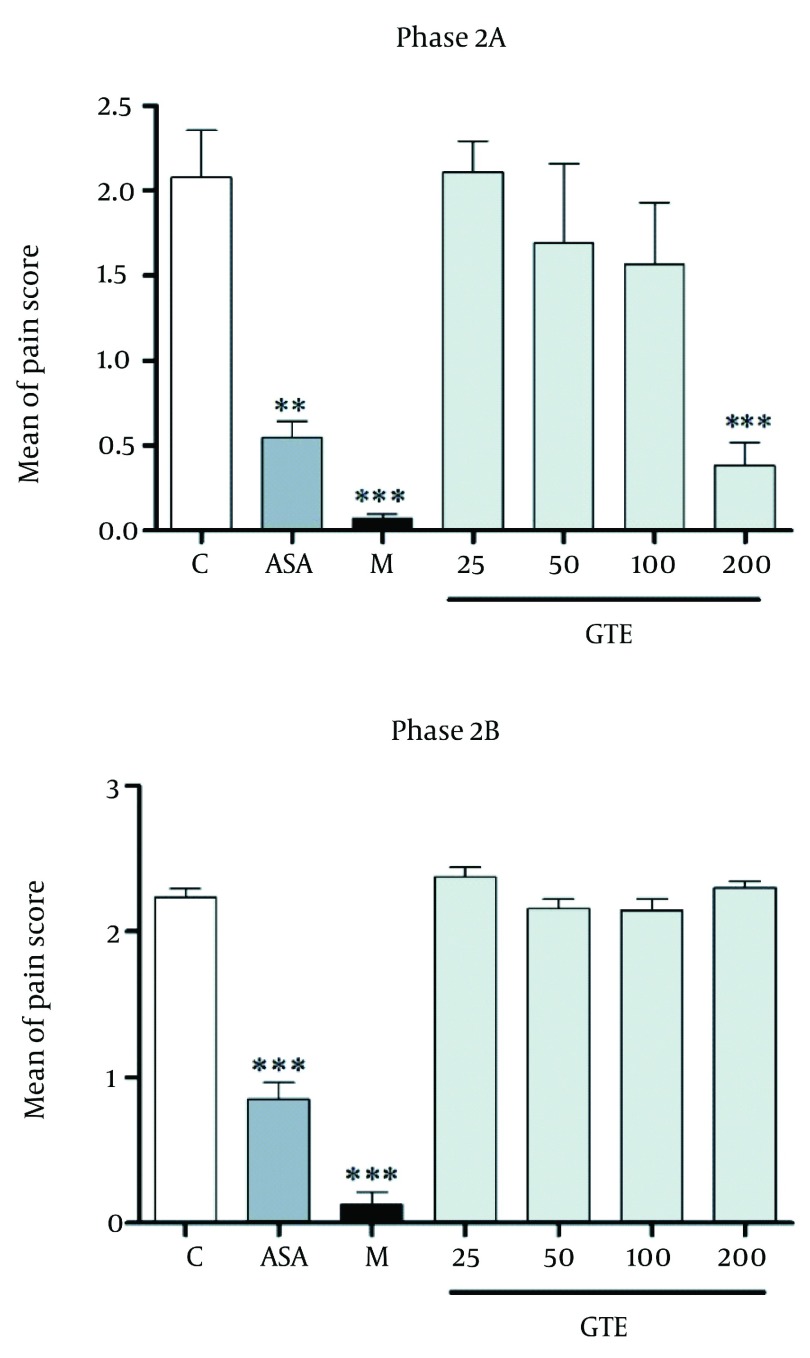
Effect of GTE (25-200 mg/kg, i.p.), morphine (2.5mg/kg, i.p.) and Aspirin (300mg/kg, i.p.) During Phase 2A and Phase 2B of the Formalin-induced Nociception in rat. Each Column Represents Mean ± SEM (n=6). ** P < 0.01 and *** P < 0.001 Compared to Control (Treated With Vehicle).

**Figure 3:**
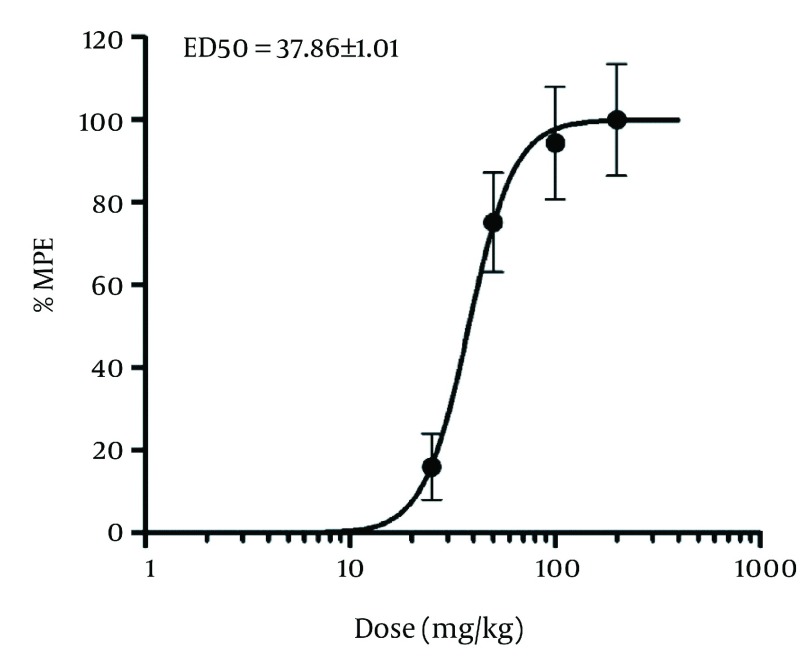
Dose-response Curve for the Antinociceptive Activity Induced by Intraperitoneal Administration of GTE, in First Phase of the Formalin Test in rats. % MPE, Antinociception Expressed as Percentage of Maximum Possible Effect.

Figure 1 indicates the effect of GTE, morphine and aspirin pretreatment on formalin-induced pain during acute phase in rats. All drug-treated groups displayed significant reduction in formalin-induced nociceptive behavior when compared with the vehicle-treated group. The intraperitoneal administration of GTE thirty minutes before the injection of formalin suppressed formalin-evoked pain behavior during phase 2A (onset of second phase) compared to control conditions, but not during phase 2B ( Figure. 2 ). Observed responses in phase 1 were dose-dependent and ED50 for phase 1 was 37.86 ± 1.01 ( Figure 3 ).

**Figure 4:**
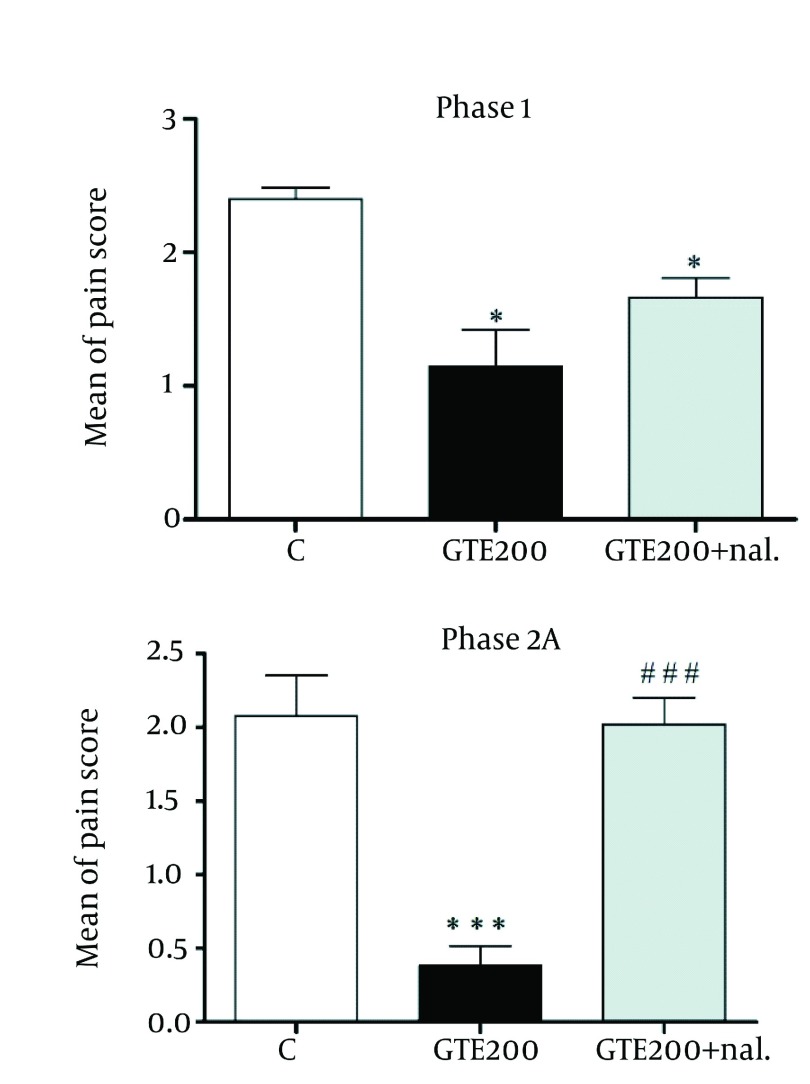
Effect of Pretreatment of Rats With Naloxone (1 mg/kg, i.p.) on the Antinociceptive Effect of GTE 200 mg/kg, i.p. Against Phase 1 and Phase 2A of the Formalin-induced Nociception Test in rat. Each Column Represents Mean ± SEM (n=6). * P <0.05 Compared to Control (Treated With Vehicle) and ### P < 0.001Compared to GT200.

The results presented in ( Figures 4 ) indicate that the pre-treatment of rats with naloxone (1 mg/kg, i.p.) significantly reversed (P < 0.001) antinociception by GTE (200 mg/kg, i.p.) on phase 2 but not on phase 1 of pain.

## 5. Discussion

The present results indicate that hydroalcoholic extract of Green tea (GTE) has antinociceptive activity in chemical pain tests. The formalin-induced paw pain, an in vivo model of persistent pain, is a valid model for analgesic study, and is believed to represent a significant model of clinical pain and very popular for the rapid and easy screening of pharmacological targets in drug evaluation. The formalin test produces a distinct biphasic nociceptive response. The first phase (neurogenic pain), occurring within seconds of formalin injection, is elicited by direct chemical activation of nociceptive primary afferent fibers. The second, later phase (inflammatory pain), occurs as a result of ongoing activity in primary afferents and increased sensitivity of dorsal horn neurons. Therefore, the test can be used to clarify the possible mechanism of antinociceptive effect of a proposed analgesic (15). Centrally acting drugs, such as opioids, inhibit both phases equally (16); however, many NSAIDs and corticosteroids inhibit only the late phase (17). Green tea is rich in flavonoids and there is much evidence accumulated that flavonoids possess important effects on various biological systems, which may explain their widespread therapeutic uses (18). Gulnur et al. (2004) reported anti-inflammatory and antinociceptive activities of main flavonoid glycosides isolated from the leaves of Tiliaargentea (19). Chaudhuri et al. (2005) showed that the Indian black tea extract could effectively reduce histamine and serotonin-induced rat paw oedema (active in the initial phase) while no significant alteration was observed against prostaglandin E2-mediated inflammation. Green tea extract at the current study inhibited both phases of the formalin test (phase 1 and 2A) but more effectively the former rather than the latter. Current study data are consistent with the observation that phase2A is more sensitive to inhibitory effects of pharmacological agents than phase 2B (11). Changes in active inhibition that produce the interphase of formalin pain behavior alter the onset of phase 2 (phase 2A), but not the offset of phase 2 (phase 2B) (20) and may contribute to the behavioral phenotype observed here. This implies that GTE is effective against both neurogenic and inflammatory pain. It could be concluded that green tea extract primarily affects the central nervous system (17). The inhibitory effect of high dose of GTE in the second phase also suggests a peripherally action of GTE. Chattopadhyay et al. (2004) reported anti-inflammatory effect of tea root extract on arachidonic acid-induced paw oedema in rats (21). Naloxone, a nonselective opioid antagonist significantly reversed the antinociceptive effect of GTE in the second phase suggesting a possible opioidergic involvement in the actions of GTE. Interestingly, the failure of naloxone to exert an antagonistic effect on the action of GTE in phase 1 of the formalin test was noticed which suggested that the peripheral antinociceptive effects of GTE might be related to opioid receptors.

In conclusion we showed that hydroalcoholic extracts of Iranian green tea possess significant antinociceptive effects in the formalin test. In addition, these data provide evidence for antinociceptive effect of Iranian green tea antagonized by naloxone, which suggests that this activity may involve the opioidergic pathways. The antinociceptive effect of the extracts may be due to their content of flavonoids and tannins. Further studies are currently underway to isolate and characterize the content of active principle(s) of Iranian green tea extract.
